# Spatial and Temporal Variations in the Occurrence and Foraging Activity of Coastal Dolphins in Menai Bay, Zanzibar, Tanzania

**DOI:** 10.1371/journal.pone.0148995

**Published:** 2016-03-02

**Authors:** Andrew J. Temple, Nick Tregenza, Omar A. Amir, Narriman Jiddawi, Per Berggren

**Affiliations:** 1 School of Marine Science & Technology, Newcastle University, Newcastle-upon-Tyne, United Kingdom; 2 Chelonia Limited, The Barkhouse, North Cliff, Mousehole, Cornwall, United Kingdom; 3 Ministry of Livestock and Fisheries, Nyangumi House, Maruhubi Street, Zanzibar, Tanzania; 4 Institute of Marine Sciences, Dar es Salaam University, Zanzibar, Tanzania; Università degli Studi di Napoli Federico II, ITALY

## Abstract

Understanding temporal patterns in distribution, occurrence and behaviour is vital for the effective conservation of cetaceans. This study used cetacean click detectors (C-PODs) to investigate spatial and temporal variation in occurrence and foraging activity of the Indo-Pacific bottlenose (*Tursiops aduncus*) and Indian Ocean humpback (*Sousa plumbea*) dolphins resident in the Menai Bay Conservation Area (MBCA), Zanzibar, Tanzania. Occurrence was measured using detection positive minutes. Inter-click intervals were used to identify terminal buzz vocalisations, allowing for analysis of foraging activity. Data were analysed in relation to spatial (location) and temporal (monsoon season, diel phase and tidal phase) variables. Results showed significantly increased occurrence and foraging activity of dolphins in southern areas and during hours of darkness. Higher occurrence at night was not explained by diel variation in echolocation rate and so were considered representative of occurrence patterns. Both tidal phase and monsoon season influenced occurrence but results varied among sites, with no general patterns found. Foraging activity was greatest during hours of darkness, High water and Flood tidal phases. Comparisons of echolocation data among sites suggested differences in the broadband click spectra of MBCA dolphins, possibly indicative of species differences. These dolphin populations are threatened by unsustainable fisheries bycatch and tourism activities. The spatial and temporal patterns identified in this study have implications for future conservation and management actions with regards to these two threats. Further, the results indicate future potential for using passive acoustics to identify and monitor the occurrence of these two species in areas where they co-exist.

## Introduction

Creation of effective conservation and management strategies for organisms is a complex and involved process. Numerous factors affect the ecology of species, yet many management strategies are initially developed with only a basic level of species and ecosystem knowledge. Detailed understanding of distribution and occurrence and how these may be affected by environmental and anthropogenic factors are perhaps the most important aspects in the creation of localised conservation and management strategies for odontocetes [1–3].

Controlling factors may act in isolation, however most controls arise through complex interactions [4,5]. Factors linked with the distribution, occurrence and behaviour of odontocetes include: prey availability [6–8]; depth and bathymetry [9,10]; tourism pressure [11–14] even with strict guidelines being followed [15,16]; fishing pressure and bycatch [17–20]; boat exposure [21,22]; time of day and diel cycle [9,23,24]; tidal patterns [25–27]; temperature and climate change [8,9,28]; season [6,8,9]; and predation risk [29].

Passive Acoustic Monitoring (PAM) allows for fine-scale study of odontocete occurrence by recording high-frequency broadband click vocalisations across large temporal scales and throughout complete diel cycles [3,30,31]. Acoustic detection effectiveness is not significantly affected by time of day, weather, visibility, odontocete surface presence, sea state, observer bias or other factors which hamper traditional visual surveys and are unlikely to affect odontocete behaviour [32]. Further, acoustic monitoring has been shown to yield up to seven times the detection rate of visual surveys [33].

Eight species of delphinid occur around Zanzibar but only the Indo-Pacific bottlenose dolphin, *Tursiops aduncus* and the Indian Ocean humpback dolphin, *Sousa plumbea* are resident in the Menai Bay Conservation Area (MBCA), located off the southwest coast of Unguja Island, year round [34,35]. Both dolphin species in the MBCA are threatened by fisheries bycatch [19,20] and are negatively impacted by boat based tourism [11,13]. Both species have low estimated population sizes, 136 (124–172 CI) *T*. *aduncus* and 63 (57–95 CI) *S*. *plumbea* [35], highlighting this vulnerability. Yet, currently no management exists for these species from a fisheries or tourism perspective. No previous research in the area has investigated variations in distribution, occurrence or behaviour over differing temporal scales. Further, there is limited understanding of impacts and constraints imposed by environmental and anthropogenic factors on the species’ distribution and ecology.

This study uses PAM to monitor and assess variations in dolphin occurrence and foraging behaviour with respect to spatial (location) and temporal (diel and tidal phases, monsoon season) factors within the MBCA. It is currently not possible to distinguish the echolocation signals for the two study species and so they are considered as a singular amalgamated population. Whilst this limits the use of results to infer species-specific information, given the ecological overlap, the shared threats and resultant survival risk mean the study provides invaluable information for the future management and conservation of both species.

## Materials and Methods

**Fig 1:**
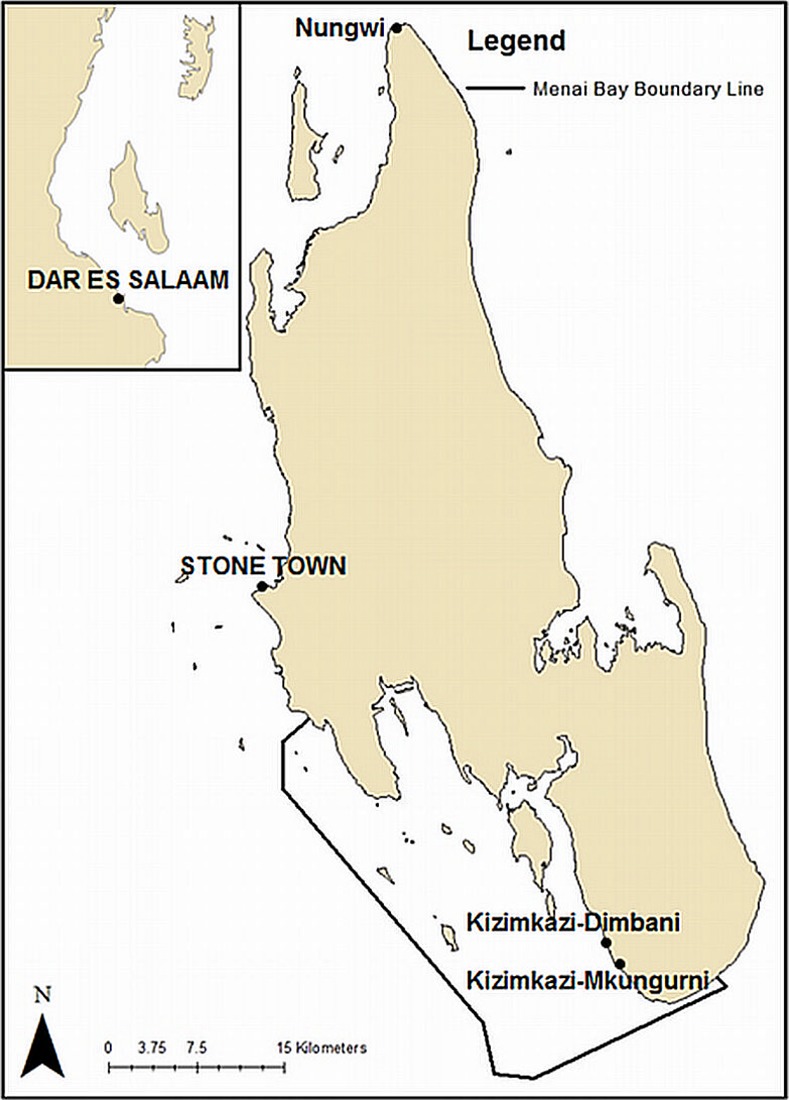
Map of Unguja Island. Unguja Island, showing the 470km^2^ Menai Bay Conservation Area (6.38°, 39.37°), the capital Stone Town and villages of Nungwi, Kizimkazi-Dimbani and Kizimkazi-Mkungurni (Source: Institute of Marine Sciences, Dar es Salaam University).

The study was carried out in the MBCA (-6.38°, 39.37°) off the southwest coast of Unguja Island, Zanzibar, Tanzania (Fig 1) between 2^nd^ February and 3^rd^ April 2013. Research was carried out under a research permit issued by the Second Vice Presidents Office of Zanzibar and processed by the Ministry of Livestock and Fisheries, Zanzibar.

The MBCA, established in 1997, is Zanzibar’s second largest marine protected area at 470km^2^. It is mostly shallow (<50m depth) soft sediment dominated with a fringing coral reef along the coastline and around the small islands, with associated seagrass beds, and a number of offshore reefs [36]. The tidal regime is semi-diurnal with large portions of reef exposed during low tide. Surface currents in the area are wind-driven and change with the two main seasons, the Kaskazi (north-easterly winds) and Kusi (south-easterly winds). During the Kaskazi the MBCA is generally sheltered from the dominant wind-driven southerly currents by the offshore islands and reefs, conversely during the Kusi season northerly currents become dominant, impacting the bay and driving an upwelling system [37]. Restrictions on the use of destructive fishing gear and foreign vessels exist in the MBCA, however enforcement effectiveness has been questioned with Tanzania mainland vessels reported to fish within the MBCA boarders.

### Acoustics

**Fig 2:**
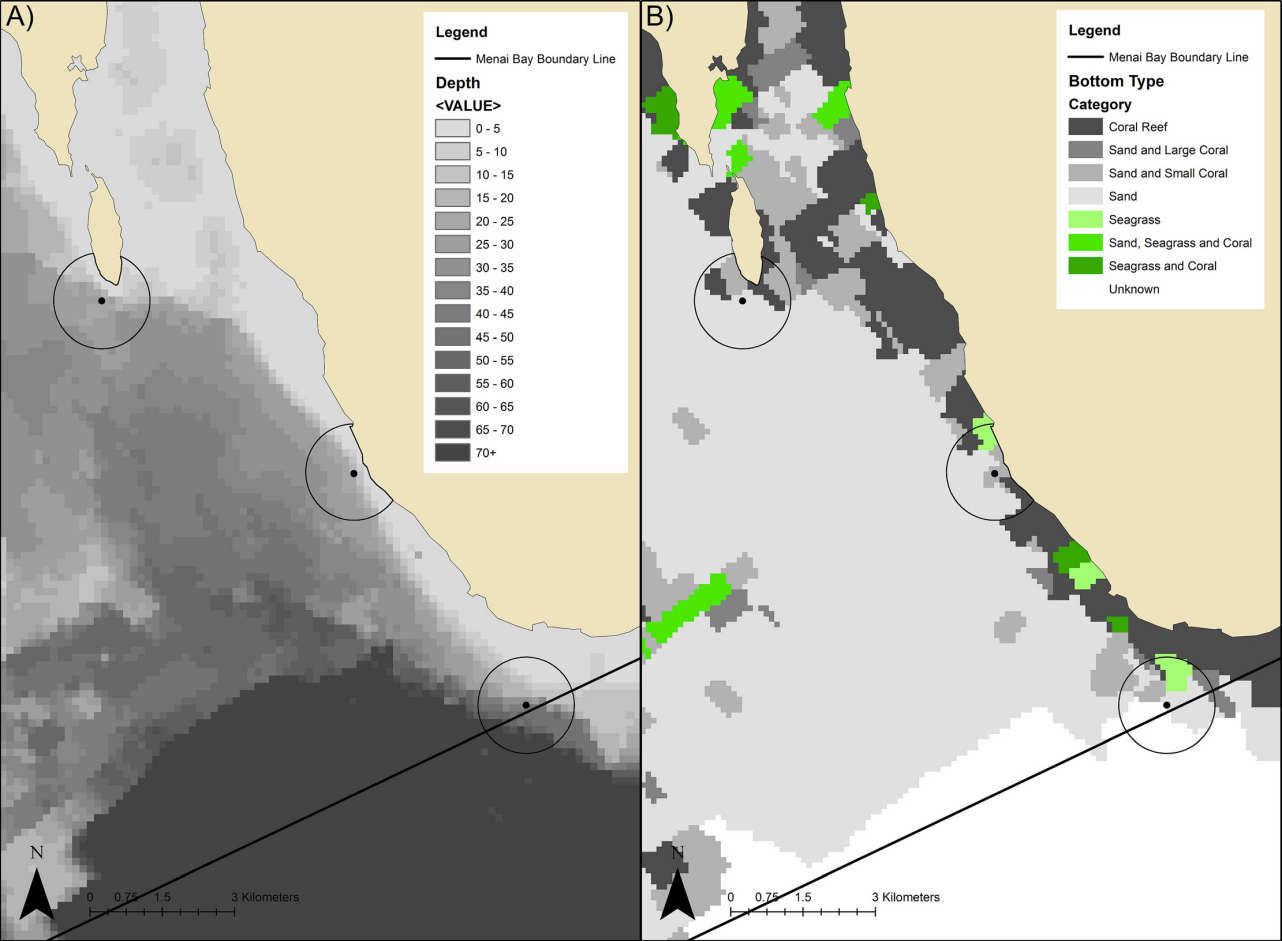
C-POD deployment site maps. (A) bathymetric map and (B) benthic cover map. C-POD deployment sites with estimated maximum detection distance of 1000m are displayed: (1) Usine–high tourism pressure, (2) L’Oasis–moderate tourism pressure, (3) Massoni–no tourism pressure, control site.

Cetacean click recorders (C-PODs, www.chelonia.co.uk) were anchored at three sites of similar depth (18m ± 0.3m) off of the fringing coral reef (Fig 2) but with differing tourism pressures [35]: (1) Usine (-6.489°, 39.493°), high dolphin tourism pressure; (2) L’Oasis (-6.445°, 39.461°), moderate dolphin tourism pressure; and (3) Massoni (-6.413°, 39.414°), no known dolphin tourism. C-PODs record clicks in the frequency range 20-160kHz and were deployed with low sensitivity settings, to exclude high levels of background noise, likely be caused by snapping shrimp (*Alpheidae sp*.). C-PODs were deployed 5m from the seabed and anchored with rope to large coralline rocks. Surface lines and buoys were not used in order to keep the equipment inconspicuous.

At L’Oasis and Massoni 1441 hours of continuous data were recorded. The Usine C-POD was removed by fishermen and later recovered; consequently only 1077 hours of continuous data were recorded up until the 20^th^ of March 2013.

Acoustic data was extracted using CPOD.exe software (www.chelonia.co.uk). Click Trains (CTs) were extracted using the Generalised Encounter Classifier (GENENC). Only high quality CTs (“Other cet”) were used. Poor correspondence between high and low quality (“Other cet?”) results indicated a high likelihood of false positives in the low quality classification. Encounters were defined as a series of CTs with gaps of less than five minutes. Ten minute gaps have been used previously [3] but were considered overly conservative given higher travel speeds observed in *Tursiops sp*. 4.3–13.9km h^-1^ [23,38] and high density of animals expected in parts of the MBCA. Detection Positive Minutes (DPM, a minute in which at least one dolphin click train is detected) were used as a proxy for relative dolphin occurrence. Series’ of Inter-Click Intervals (ICIs) of <9ms were used to identify terminal buzz vocalisations [39–41] indicative of foraging behaviour [42,43]. Terminal Buzz Positive Minutes (BPM, a minute in which at least one terminal buzz vocalisation is detected) were used to analyse changes in foraging behaviour, as has been done in other studies [44]. ICIs were identified with “high quality ICI” filters only.

Maximum detection has been estimated to be between 800-1246m for *T*. *truncatus* in Timing Porpoise Detectors (T-PODs) [30,45] and 1343-1779m in C-PODs, with a median detection range of 462-729m [46]. In this study it was not possible to estimate maximum detection distance for *T*. *aduncus*, but 1000m was used as an approximate detection distance when considering environmental and anthropogenic variables that may influence occurrence. Sites were spaced approximately 6km apart ensuring that maximum detection distances did not overlap.

Though species separation using acoustic data has been achieved in some cases [47,48], separation of *T*. *aduncus* and *S*. *plumbea* has not yet been achieved. Both species likely produce echolocation clicks following a characteristic delphinid pattern. Vocal range and idealised click spectrum for *Tursiops sp*. is well documented [49] but relatively little is known of *T*. *aduncus* [50]. Neither vocal range nor broadband click spectrum have been mapped for *S*. *plumbea*, however these have been described in both wild [24,51–53] and captive [54,55] *S*. *chinensis*, with broadband click spectra appearing similar to those of other delphinids. For the purpose of this study both species are treated as a single population. However, the study makes initial efforts to infer differences in species vocalisation behaviour based on CT sound frequencies (modal kHz–the modal value of the frequency of all individual clicks in a train) data and existing species distribution knowledge.

### Temporal Variables

Data were analysed with respect to three temporal variables: monsoon seasonality, diel phase and tidal phase. To permit comparisons between dolphin occurrence rates in each season, the study period covered both the end of the Kaskazi, north-easterly winds (November–March/April), and the beginning of the Kusi, south-easterly winds (March/April–October), monsoon seasons. The Kusi began unusually early on the 8th of March 2013. Diel phase was defined as Sunrise (sunrise time ± 30mins), Sunset (sunset time ± 30mins), Day (between Sunrise and Sunset) and Night (between Sunset and Sunrise). Tidal phases were defined at High (high tide ± 60mins), Low (low tide ± 60mins), Ebb (between High and Low) and Flood (between Low and High) as seen in previous works [25,56]. Tide time data for Zanzibar was obtained from Mobile Geographics (www.tides.mobilegeographics.com).

### Other Variables

Limited bathymetric and benthic cover data were available for the MBCA. Data were provided to address this [57] and updated maps are presented (Fig 2). Data were processed and visualised using ArcGIS 10.1 software (www.esri.com). The bathymetric map (Fig 2A) was produced using Inverse Distance Weighted (IDW) interpolation. Benthic cover was defined as a set of discrete classifications; therefore an IDW was not appropriate for these data. A gridded overlay was created (100m x 100m) and projected onto the study area. Grid cells were populated by classification, assigning the benthic type (Reef; Sand and Large Coral; Sand and Small Coral; Seagrass; Sand and Seagrass; Sand, Seagrass and Coral; Sand; Mud: Unknown) of the closest known point to the cell’s centre using a spatial join with “match option” set to “closest” (Fig 2B). Limited resolution means fine-scale errors may exist. Despite limitations, the map matches known features and was verified by local fishers.

**Fig 3:**
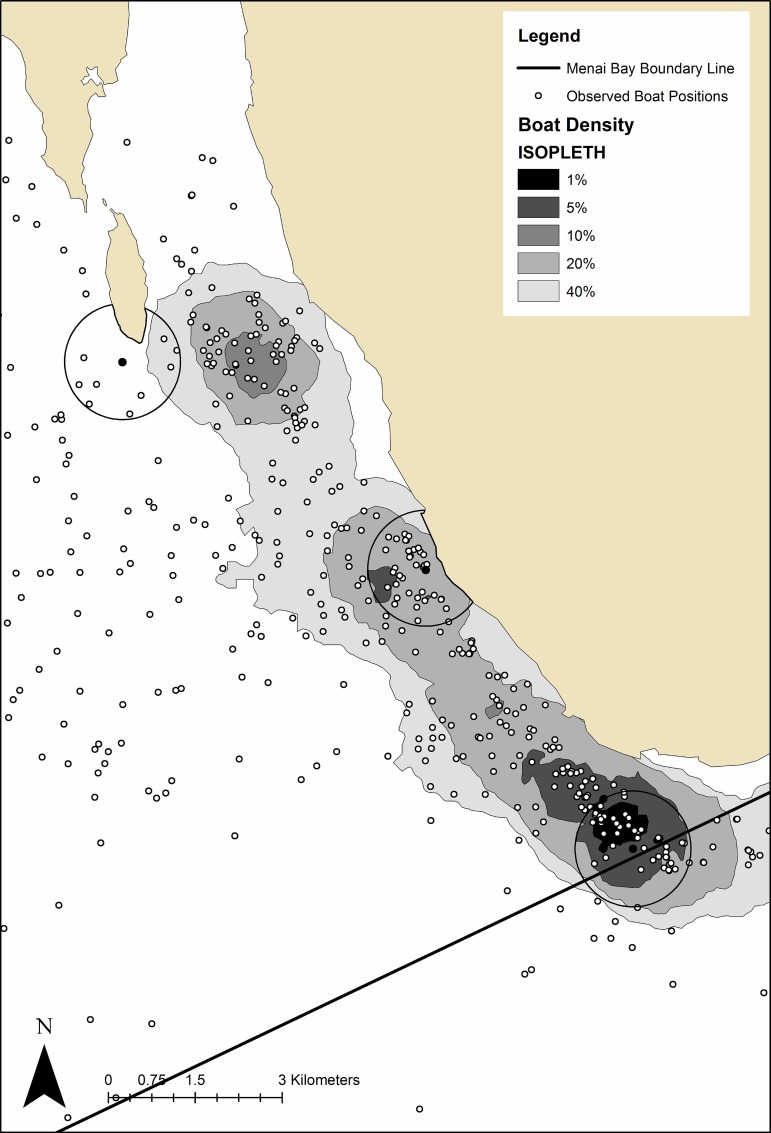
Menai Bay Conservation Area boat density map. Isopleth map showing areas of highest boat density. Total number of boats sighted at (1) Usine = 57 (25 Fishing, 21 Tourism), (2) L’Oasis = 37 (17 Fishing, 3 Tourism) and (3) Massoni = 9 (3 Fishing, 0 Tourism). C-POD deployment sites with 1000m detection radius are displayed.

Boat sightings data were also provided, collected during distance sampling transect surveys [57]. Data were processed in ArcGIS 10.1 and isopleths were generated using Geospatial Modelling Environment (GSM, www.spatialecology.org) to determine areas of highest observed boat density (Fig 3). Most data were collected between 6am and 4pm and so results are only representative of this time period. Peak drift netting activity occurs between 7pm and 5am, longlining and handlining between 3am and 12pm and set gillnets are present continuously. Dolphin tourism occurred exclusively during daylight hours. Data were therefore representative of dolphin tourism pressure and assumed to be representative of overall fishing pressure. Relative tourism pressure recorded here corresponded closely to patterns reported in previous work [35].

### Analysis

Minitab 17 was used for all statistical analyses. Normality was assessed with Kolmogorov-Smirnoff and equal variance assessed with Levene’s test as appropriate. All post-hoc tests p-values were corrected using a Bonferroni correction where appropriate.

Data were compiled and modelled in relation to spatial (location) and temporal (diel phase, tidal phase and monsoon season) variables. Available environmental and anthropogenic variables (primary productivity, prey density, benthic composition, tourism pressure and boat density) were excluded from this analysis as they could not be considered independent of study site or data were not available to an appropriate degree of detail within the confines of this study. Sea surface temperature, obtained from the Natural Environment Research Council (NERC) Earth Observation Data Acquisition and Analysis Service (NEODAAS), was omitted after an average variation of less than 0.4°C was found across the different sites over the entire study period and was considered to be biologically irrelevant. Temperature at depth could not be used in this analysis as the damage sustained by the Usine C-POD did not allow for retrospective calibration of the internal thermometers and so direct comparisons between sites could not be made. Models were not expected to explain a high proportion of the overall variability in DPM for a number of reasons: the low number of variables that could be included in the modelling process; the likelihood that not all occurrences of dolphins within the study area would be recorded by the C-POD, with detection probability reducing with distance; and the overall low probability of DPMs. This also limited the need for more advanced data modelling methods.

Binomial Logistic Regression models were used to investigate the significance of variables in predicting occurrence and foraging activity, with DPM and BPM as categorical binary response variables where appropriate. Stepwise backwards elimination was used to remove non-significant (p > 0.05) variables from the model.

In order to assess the effects of variables on BPM occurrence only data from DPM were assessed, thus removing bias created by the effects of variables on DPM occurrence. When assessing BPM against temporal variables low BPM frequencies during Sunset and Sunrise diel periods resulted in quasi-complete separation of data and prevented the running of the model. As such when analysing BPM against diel phase Sunrise and Sunset were excluded as factors from the analysis.

Chi-Squared tests were used to assess differences in DPM frequency between sites during specific diel phases, BPM frequency between sites and between diel phases within sites. Frequency analysis rather than the model was used to compare BPM between sites as a higher probability of BPM during DPM does not necessarily reflect the overall importance of an area for foraging.

Encounter length was measured as DPM Encounter^-1^. Average encounter lengths were compared between sites in order to look for any implications of differences in behaviour displayed between areas, reasoning that low average encounter lengths are indicative of dolphins exhibiting a lower proportion of geographically static behaviours (i.e. foraging, resting) and a higher proportion of travelling behaviours.

Previous studies suggest changes in apparent odontocete occurrence resulting from diel variation in vocalisation rate [58–60]. Echolocation rate was proxied as Clicks DPM^-1^, reasoning that echolocating at a higher rate would produce a greater average number of clicks. Average Clicks DPM^-1^ were compared between diel phases within sites to assess any potential influence and bias on occurrence.

Reported differences in the proportional representation of species at each site [35], Usine and L’Oasis in particular, allowed for an initial investigation of differences in broadband click spectra. The pattern of broadband CT sound frequencies was examined graphically and implications for the broadband click spectra of *T*. *aduncus* and *S*. *plumbea* are discussed.

### Ethical Approval

Ethical approval for this study was granted by the Science, Agriculture and Engineering Ethical Committee and by the Animal Welfare and Ethical Review Body of Newcastle University.

## Results

**Fig 4:**
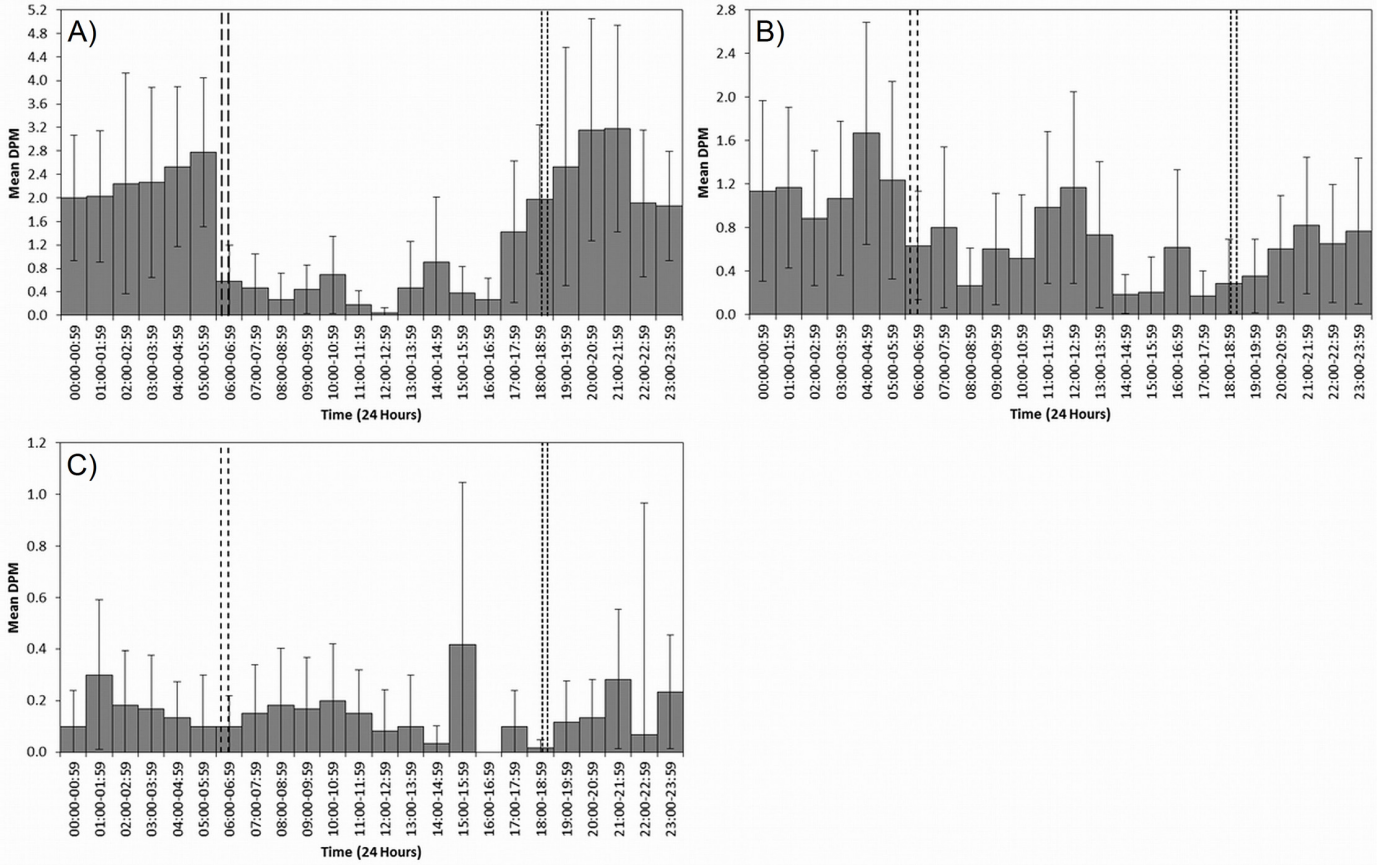
Occurrence in relation to time of day. Mean Detection Positive Minutes (DPM) per hour bins for the whole study period at (A) Usine–high dolphin tourism, (B) L’Oasis–moderate dolphin tourism and (C) Massoni–no dolphin tourism (control). 95% mean confidence interval error bars are displayed as a measure of variance. Large and small dashed lines represent sunrise and sunset time ranges for the study period, respectively.

C-PODs at L’Oasis and Massoni logged data for the entire study period. The Usine C-POD was removed by fishermen and later recovered; consequently data for this site is not available for the entire study period. An overview of the data (Table 1) and mean DPM by hour of day (Fig 4) are presented.

**Table 1:** Summary of study period and C-POD data.

Category	Site		
	Usine	L’Oasis	Massoni
Start Date	02/02/2013	02/02/2013	02/02/2013
End Date	20/03/2013	03/04/2013	03/04/2013
Recording (Days)	44	60	60
Recording (Hours)	1077	1441	1441
Encounters	256	172	64
Detection Positive Minutes	1556	1049	211
Clicks	113830	80529	14732
Click Trains	10955	7641	1131
Buzz Positive Minutes (High Quality)	58 (26)	41 (20)	18 (11)

### Spatial and Temporal Influences on Occurrence

Significant effects of spatial and temporal variables on probability of occurrence were found (Binomial Logistic Regression, χ^2^ = 2216.11, df = 9, p < 0.001) accounting for 7.22% of variation. Site was the most significant variable (χ^2^ = 1605.78, p < 0.001), with probability of DPM at Usine increased by 890% (OR = 10.12) and 99% (OR = 2.01) from Massoni and L’Oasis respectively, with the probability of DPM at L’Oasis 398% (OR = 5.30) greater than at Massoni. Diel Phase (χ^2^ = 601.74, p < 0.001) and Tidal Phase (χ^2^ = 9.52, p < 0.05) were also significant. Monsoon season did not appear to have any effect on the probability of detections (χ^2^ = 0.67, p = 0.412), suggesting they do not impact on overall dolphin occurrence in the MBCA. However, as the spatial variable was the most significant factor it was not appropriate to draw conclusions on the influence of temporal variables across sites from this model. Therefore we re-analysed the effect of temporal variables separately for each site. Subsequent Logistic Regression Models were run for each site following the same procedure.

At Usine significant impacts were seen for both diel and tidal phase variables. There was no evidence for effects of monsoon season, though reduced sampling in the Kaskazi due to the early removal of the C-POD may compromise the validity of this result. There was no evidence for differences in DPM probability between Low, Ebb and Flood tidal phases and DPM frequencies showed no differences between these phases (Chi-Squared, χ^2^ = 1.9 df = 2, p > 0.05). These tidal phases were grouped and the model re-run. The improved model showed significant impacts of diel and tidal phases on DPM probability (Table 2) (Binomial Logistic Regression, χ^2^ = 729.0, df = 4, p < 0.001) accounting for 4.94% of variation. Diel phase was most significant (χ^2^ = 718.37, p < 0.001) with the probability of detections higher during Night compared to all other phases, Sunset higher than Sunrise and Day, but no evidence for any differences between Sunrise and Day. The increased probability of DPM at Night over Day may suggest a diel phase mediated difference in echolocation rates, as has been suggested in other works. However if this was the only contributing factor differences in Sunset and Sunrise occurrence would not be expected. DPM probability was increased during High tide compared to other tidal phases, the difference is relatively small but could reflect some level of restricted access to the Usine site during other tidal phases.

**Table 2:** Binomial Logistic Regression Models outputs for temporal variables which have significant impact on the probability of dolphin occurrence (Detection Positive Minutes). OR = Odds Ratio.

Site	Comparisons	OR (95% CI)	Significant (Y/N)	Detection Probability Change (based on OR)
**Usine**	**Diel Phase**			
	Night v Day	4.91 (4.28–5.63)	Y	+375%
	Sunrise v Day	1.12 (0.75–1.69)	N	n/a
	Sunset v Day	3.92 (3.06–5.00)	Y	+282%
	Sunrise v Night	0.23 (0.16–0.34)	Y	-76%
	Sunset v Night	0.80 (0.65–0.99)	Y	-20%
	Sunset v Sunrise	3.49 (2.24–5.41)	Y	+240%
	**Tidal Phase**			
	Other v High	0.82 (0.72–0.94)	Y	-17%
**L’Oasis**	**Diel Phase**			
	Night v Day	1.67 (1.47–1.90)	Y	+66%
	Sunrise v Day	1.09 (0.78–1.52)	N	n/a
	Sunset v Day	0.48 (0.30–0.79)	Y	-51%
	Sunrise v Night	0.65 (0.47–0.91)	Y	-35%
	Sunset v Night	0.29 (0.18–0.47)	Y	-71%
	Sunset v Sunrise	0.44 (0.25–0.79)	Y	-55%
	**Tidal Phase**			
	Flood v Ebb	1.07 (0.93–1.23)	N	n/a
	High v Ebb	0.85 (0.71–1.03)	N	n/a
	Low v Ebb	0.58 (0.47–0.72)	Y	-41%
	High v Flood	0.80 (0.66–0.96)	Y	-20%
	Low v Flood	0.55 (0.44–0.68)	Y	-45%
	Low v High	0.68 (0.53–0.88)	Y	-31%
	**Season**			
	Kusi v Kaskazi	1.19 (1.05–1.34)	Y	+19%
**Massoni**	**Tidal Phase**			
	Flood v Ebb	0.49 (0.33–0.73)	Y	-51%
	High v Ebb	1.19 (0.82–1.73)	N	n/a
	Low v Ebb	1.49 (1.05–2.10)	Y	+48%
	High v Flood	2.42 (1.56–3.74)	Y	+141%
	Low v Flood	3.02 (2.00–4.58)	Y	+202%
	Low v High	1.25 (0.84–1.87)	N	n/a
	**Season**			
	Kusi v Kaskazi	0.62 (0.47–0.83)	Y	-38%

At L’Oasis DPM probability was significantly effected by diel and tidal phases and monsoon season (Table 2) (Binomial Logistic Regression, χ^2^ = 131.32, df = 7, p < 0.001) accounting for 1.10% of the variation. Diel phase was the most significant variable (χ^2^ = 87.63, p < 0.001) with the probability of DPM increased significantly at Night compared to all other phases, increased during the Day and Sunrise compared to Sunset with no significant difference between Sunrise and Day probabilities. Similarly to Usine the increased DPM at night may suggest some effect of echolocation rates yet the differences between Sunrise and Sunset would not be expected if this was a sole contributing factor. Tidal phase influence (χ^2^ = 37.70, p < 0.001) was also distinct, with DPM significantly lower during Low tide than all other phases, High and Flood tides did not significantly vary from Ebb tides but DPM probability at Flood was significantly higher than at High tide. Initially lower DPM probability during Low tide may indicate reduced accessibility to the L’Oasis site, however the significantly increased DPM probability at Flood and non-significantly increased probability during Ebb over High tide suggests this is not the sole reason for differences in this area. Lastly monsoon season exerted a significant effect (χ^2^ = 7.67, p < 0.01), with increased DPM probability during the Kusi season, contrary to the results at Usine.

At Massoni DPM probability was significantly affected by tidal phase and monsoon season (Table 2) (Binomial Logistic Regression, χ^2^ = 42.96, df = 4, p < 0.001) accounting for 1.32% of the variation. Unlike the other sites diel cycle did not have any effect, this may have implications for the use of the area by dolphins. The patterns of tidal phase (χ^2^ = 32.22, p < 0.001) effects differed from both Usine and L’Oasis. Flood tidal phase showed significantly reduced DPM probability compared to all other phases. Low tide showed increased DPM probability from Ebb but not from High, with no significant variation between High and Ebb. The inconsistency of tidal influence across all sites suggests there is no generalised tidal affect on occurrence in coastal areas of the MBCA. Similarly the effects of monsoon season (χ^2^ = 11.04, p < 0.05) differed from other locations, with increased DPM probability during the Kaskazi, suggesting there is no general pattern in DPM linked with monsoon season as was suggested in the initial models which combined all sites.

Differences in DPM were found among sites during the day (Chi-Squared, χ^2^ = 99.3, df = 2, p < 0.001) and the night (Chi-Squared, χ^2^ = 60.9, df = 2, p < 0.001), with the highest at Usine (Day: 249 DPM, Night: 1192 DPM), followed by L’Oasis (Day: 236 DPM, Night: 457 DPM) and then Massoni (Day: 76 DPM, Night: 99DPM). However, post-hoc Chi-squared showed no difference in occurrence frequency during the day between Usine and L’Oasis (Chi-Squared, χ^2^ = 0.35, df = 2, p > 0.05). This may indicate a change in the daytime distribution of dolphins from those previously described.

Median encounter length (DPM Encounter^-1^) differed among sites (Kruskal-Wallis, H = 27.0, df = 2, p < 0.001). Post-hoc Mann-Whitney U-Tests showed no difference in encounter length between Usine (median = 4, range = 1–47) and L’Oasis (median = 5, range = 1–24) (Mann-Whitney U-Test, U = 3.8x10^4^, n_1,2_ = 256, 172, p > 0.05), but both showed longer encounter length than Massoni (median = 3, range = 1–20) (Mann-Whitney U-Test: Usine, U = 7320, n_1,2_ = 256, 64, p < 0.001. L’Oasis, U = 5240, n_1,2_ = 172, 64, p < 0.001). Shorter encounter lengths at Massoni may be indicative of a comparatively higher proportion of travelling behaviour being displayed in the site.

### Spatial and Temporal Influences on Foraging Behaviour

There were differences in BPM frequency among sites, when all C-PODs were active (Chi-Squared test, χ^2^ = 6.96, df = 2, p < 0.05). Usine had the highest number of events (26 BPM), followed by L’Oasis (15 BPM) and Massoni (11 BPM). This suggests that Usine is the most important foraging area for MBCA dolphins. Further, analyses of total BPM across the entire study period showed BPM frequency to be greater during Night at all sites, Usine (Day: 4 BPM, Night: 21 BPM) (Chi-Squared test, χ^2^ = 11.6, df = 1, p < 0.001), L’Oasis (Day: 5 BPM, Night: 15 BPM) (Chi-Squared test, χ^2^ = 5.0, df = 1, p < 0.05) and Massoni (Day: 2 BPM, Night: 9 BPM) (Chi-Squared test, χ^2^ = 4.5, df = 1, p < 0.05). This suggests that in-shore areas of the MBCA are more commonly used for foraging during night time hours. However the pattern may also reflect the apparent increase in occurrence during Night time hours across some sites and so is not necessarily representative of foraging activity patterns throughout the bay. Temporal variables influencing BPM probability within DPM were investigated.

At Usine tidal phase was the only significant variable (Binomial Logistic Regression, χ^2^ = 24.31, df = 3, p < 0.001) and accounted for 4.25% of the variation. BPM probability was greater at Flood than Ebb and Low, with no difference from High, greater at High than Low, with no difference from Ebb, and no difference between Ebb and Low (Table 3). It should be noted that while the difference between Flood and High is non-significant it appears likely that this would change with increased sample size. Significance of tidal phase suggests that it may have some impact on prey availability or foraging effectivness.

**Table 3:** Binomial Logistic Regression Models outputs for temporal variables which have significant impact on the probability of foraging behaviour (Terminal Buzz Positive Minutes). OR = Odds Ratio.

Site	Comparisons	OR (95% CI)	Significant (Y/N)	Detection Probability Change (based on OR)
**Usine**	**Diel Phase**			
	Flood v Ebb	3.54 (1.74–7.22)	Y	+237%
	High v Ebb	1.87 (0.78–4.46)	N	n/a
	Low v Ebb	0.40 (0.09–1.86)	N	n/a
	High v Flood	0.53 (0.26–1.05)	N	n/a
	Low v Flood	0.11 (0.03–0.48)	Y	-88%
	Low v High	0.22 (0.05–0.98)	Y	-78%
**L’Oasis**	**Diel Phase**			
	Night v Day	2.95 (1.29–6.73)	Y	+184%

At L’Oasis diel phase was the only significant variable (Binomial Logistic Regression, χ^2^ = 11.92, df = 1, p < 0.05) and accounted for 2.36% of the variation. It is important to note that whilst tidal phase was non-significant its potential impacts followed the same pattern as seen at Usine, with increased probability at Flood and High tidal phases. BPM probability increased significantly during Night hours compared to Day (Table 3). This suggests foraging accounts for a greater proportion of behaviours at L’Oasis during Night.

No effects on BPM were found for temporal variables at Massoni (Binomial Logistic Regression, χ^2^ = 1.42, df = 1, p > 0.05).

### Echolocation Rate

**Fig 5:**
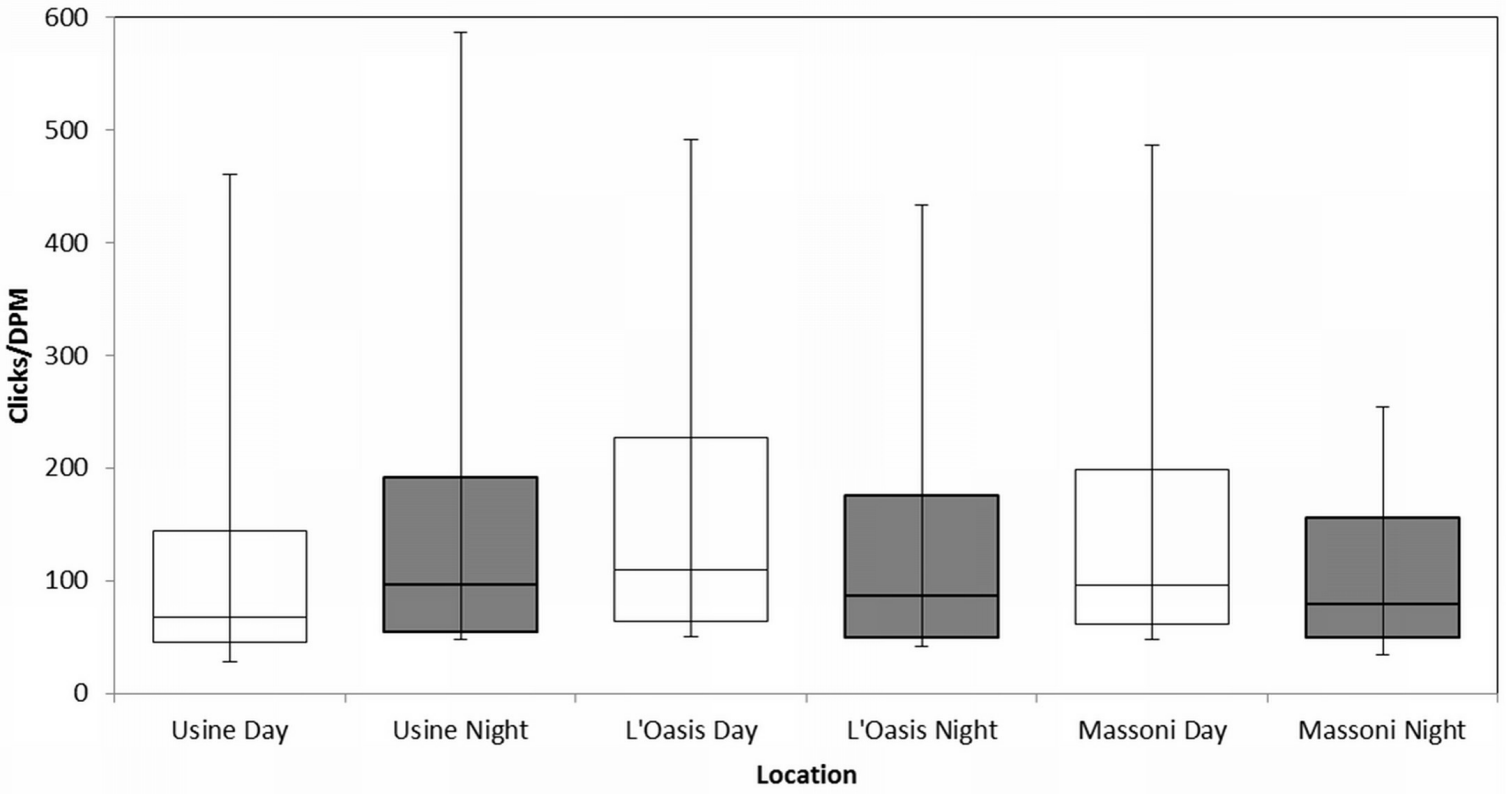
Echolocation rate between diel phases and among sites. Median clicks per Detection Positive Minute (DPM) for all study sites with interquartile range displayed. Range error bars are displayed as a measure of variance. Significant differences are found between day and night at each study site (p < 0.05).

No differences in mean echolocation rate (Clicks DPM^-1^) were found among sites (ANOVA, F = 1.86, df = 2, p > 0.05). However, median Clicks DPM^-1^ differed with diel phase within sites (Fig 5). Clicks DPM^-1^ were higher during the day at L’Oasis (Mann-Whitney U-Test: U = 2.0x10^5^, n1,2 = 110, 51, p < 0.05) and Massoni (Mann-Whitney U-Test: U = 1.0x10^4^, n1,2 = 34, 26, p < 0.05) and at night at Usine (Mann-Whitney U-Test, U = 1.4 x10^5^, n1,2 = 249, 1192, p < 0.001). Differences in echolocation rate may effect the interpretation of occurrence data, however the magnitude of difference between diel periods was small.

### Vocalisation Activity—Click Frequencies

**Fig 6:**
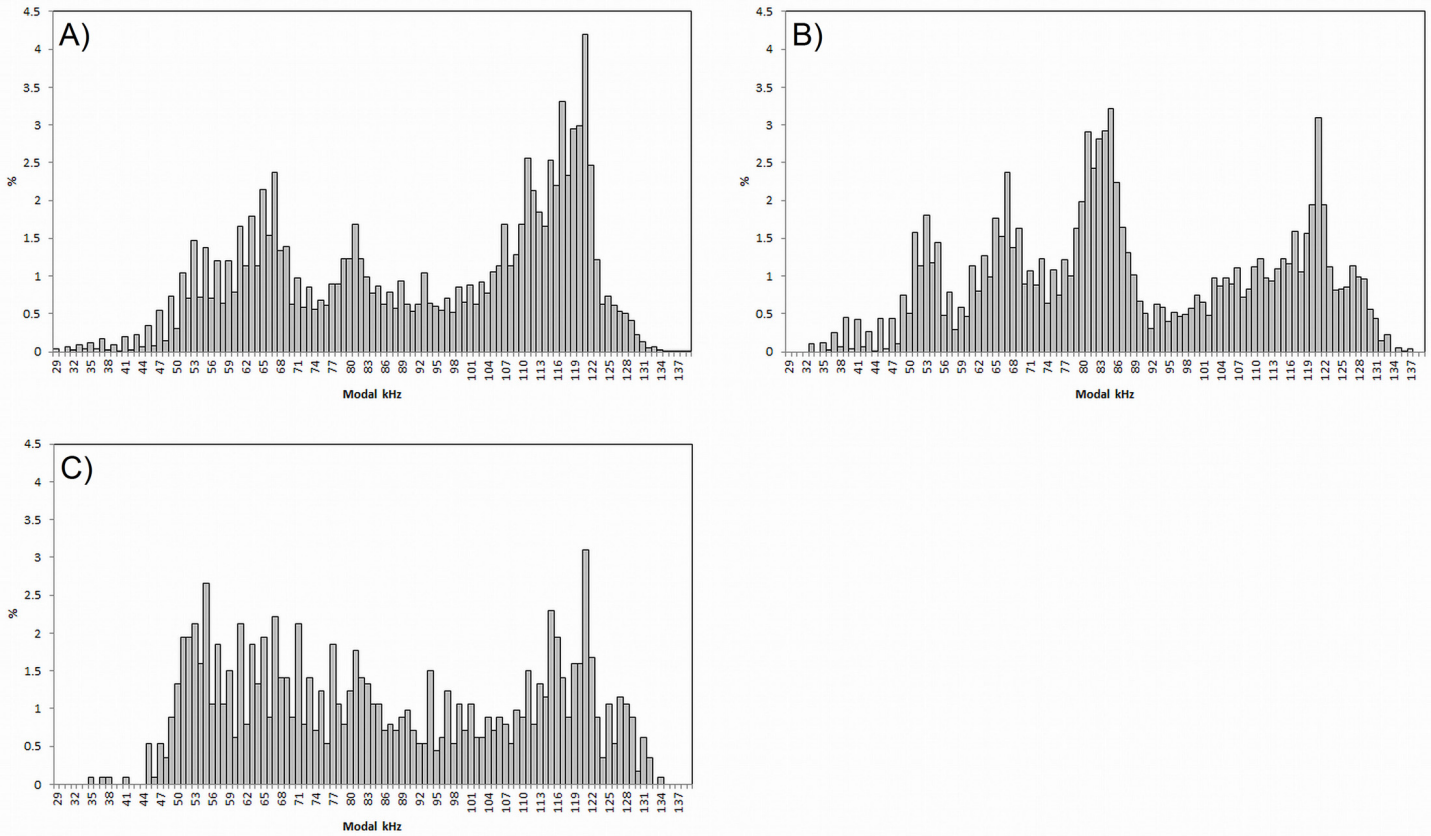
Broadband click frequencies distribution for all sites. Click Train (CT) sound frequency (modal kHz) percentage distribution for study sites across the whole study period. (A) Usine (n = 10955)–high dolphin tourism, (B) L’Oasis (n = 7641)–moderate dolphin tourism and (C) Massoni (n = 1131)–no dolphin tourism (control).

A histogram displaying the distribution CT sound frequencies was plotted for all sites to investigate differences (Fig 6). Differences in frequency patterns and in particular the frequency peaks displayed at each site may indicate a difference in the broadband click signatures of species.

## Discussion

This study is the first to describe dolphin occurrence and behaviour within the MBCA independent of human observers. It is also the first to demonstrate changes in occurrence and foraging behaviour of MBCA dolphins throughout the diel cycle and in relation to other temporal variables. Further, the work has allowed for identification of distinct patterns in dolphin occurrence at important tourism areas as well as providing improved bathymetric, benthic cover and boat density maps.

Data were modelled in respect to spatial (location) and temporal variables (seasonality, diel phase and tidal phase). Models explained relatively little of the variation in the data, although this is not surprising given the overall low probability of dolphin occurrence and the lack of data at an appropriate scale for most of the anthropogenic and environmental factors that may impact dolphin occurrence. However, significant influences of spatial and temporal variables over occurrence were found.

Location was more important than any temporal variable in predicting dolphin occurrence within the MBCA. Occurrence was highest at Usine, followed by L’Oasis and lowest at Massoni. The result supports generalised distribution patterns described in previous studies [35]. However, this previous work describes dolphin distribution only during daylight hours. In contrast, this study showed similar levels of occurrence between Usine and L’Oasis areas during daylight hours. Anecdotal evidence exists suggests a decrease in density of dolphins in the most southern areas of the bay (Usine). The results presented here suggest that whilst overall occurrence of dolphins is still highest in the southern coastal areas a partial distributional shift away from this area during the day may have occurred, indicating displacement from previously favoured habitats. Dolphin displacement may be a result of the increasing tourism pressures [13] which are highest at Usine (Fig 3), as have been observed in other geographical areas [22].

Temporal variables, tidal phase and diel phase, also exert significant effects on dolphin occurrence in the MBCA when all sites were considered together. However, as site is the most important of the explanatory variable subsequent models were run for each site individually, taking temporal variables into account.

Diel phase was the most significant temporal variable to influence occurrence of dolphins at the Usine and L’Oasis sites (Table 2), with probability of occurrence at Night significantly higher than that of Day, Sunrise and Sunset, though patterns in occurrence between other phases differed at each site. Despite the increased number of DPM at night at Massoni diel phase was not observed to exert significant influence on occurrence, though this could be the result of lower sample size at this site. In previous works differences in occurrence between diel phases have been partially or wholly attributed to changes in the echolocation rate of odontocetes [58,60]. Clicks DPM^-1^ were used as a proxy for echolocation rate, reasoning that with increased echolocation rate the average number of clicks per detection should increase. Whilst differences are found between Day and Night Click DPM^-1^ within sites, the pattern of differences were not consistent across sites and the magnitude of differences were small. Further, if echolocation rate variation was in response to different light levels it would be expected that occurrence should be similar between sunset and sunrise times. Since this is not the case we interpret the observed variation in detection rates to represent of difference in dolphin occurrence between diel phases. Therefore, the results suggest displacement of animals from Usine and L’Oasis sites during hours of daylight. One possible explanation is a distributional shift towards reef areas located offshore of the Usine and L’Oasis sites, where dolphin groups have been sighted during daylight hours [57]. A number of environmental and anthropogenic factors may drive and explain such shifts in distribution and data on these factors should be recorded in future to allow testing of their respective influences on dolphin distribution and occurrence.

Examination of DPM over time at the Usine and L’Oasis sites (Fig 4) indicated a closer correspondence of occurrence variations with the known times and magnitude of tourism activities than with sunset and sunrise times. MBCA dolphin tourism operates only during hours of daylight and is greatest between sunrise and mid to late afternoon. Decreases in dolphin occurrence at L’Oasis and Usine at 06:00–06:59 coincide with both sunrise and initiation of tourism activity. However, increases in dolphin occurrence in the afternoon coincide more closely with reduced tourism activity, first at Usine and then at L’Oasis, than with sunset. As apex predators MBCA dolphins likely exert influence on ecosystem health. If tourism is causing a distributional shift away from these areas this may have implications for both ecosystem health and the dolphin tourism industry itself, which could compromise this important alternative livelihood for locals [36].

Tidal effects on dolphin movement and distribution patterns have been observed elsewhere [26,27,61]. Tidal phase is seen to significantly influence occurrence probability across all sites. The results from this study suggested an increased probability of dolphin occurrence at Usine during High tide compared to all other tidal phases which also corresponds to anecdotal observations by local fishermen. However there was no consistent pattern across all sites, suggesting that tidal influence is likely site specific rather than having a singular tidally influenced regime. Season had an effect on occurrence probability at the L’Oasis and Massoni sites, but not at Usine. The lack of a generalised seasonal effect suggests that seasonal influence on dolphin occurrence and density was negligible during the course of this study. It is important to recognise that the temporal limitations of the study may mask true seasonal variations and a more long term monitoring study is required to investigate this properly.

The results of this study further provided insight on dolphin foraging activity among the three sites. BPM frequency suggests that Usine was the most important foraging area for MBCA dolphins of those sites monitored, followed by L’Oasis. BPM were also higher at Night at these inshore sites, with no evidence for crepuscular feeding peaks as seen in other areas [56,62]. Prey availability exerts significant direct control over dolphin distribution [5–8] and may help to explain these patterns. It is thought that prey availability may be higher in the southern coastal areas of the MBCA and the patterns of fishing boat density illustrated in Fig 3 corroborate this, assuming that fishing boat density corresponds to prey availability, with fishing activity also known to be concentrated in this region at night. Whilst conclusions cannot be drawn on foraging activity throughout the MBCA, the findings suggest that dolphins are primarily feeding nocturnally in these coastal sites. If dolphins are primarily nocturnal predators then potential similarities in foraging strategies and prey choice of MBCA *T*. *aduncus* and those of the known northern Zanzibar populations may exist [20]. Interestingly exploratory models suggested that foraging behaviour takes up a greater proportion of time (probability of BPM per DPM) during Flood tidal phases and lesser proportion during Low tide at Usine with the same pattern observed at L’Oasis, although it was non-significant. These data could suggest tidal and diel mediated foraging intensity in the MBCA, however the low sample sizes restrict the power of these analyses and so caution should be taken with the interpretation of these results.

Alongside foraging activity, average encounter lengths may give some indication in regard to area usage. Greater encounter lengths (DPM Encounter^-1^) in the Usine and L’Oasis sites may indicate a greater proportion of stationary behaviours in these sites. This reflects the higher levels of foraging activity at these areas compared to Massoni but may also indicate that these areas are used for socialising and/or resting sites. However, the extent of this acoustic study is insufficient to draw conclusions regarding socialising and resting activities, which would require support from visual observational data or a method to reliably assign the acoustic data to identify such behaviours.

In many coastal areas two or more odontocete species co-exist and detailed descriptions of the acoustic signatures is lacking for many species, preventing identification of species specific patterns in occurrence from acoustic data. In this study we make initial exploratory steps towards potential future species separation of the Indo-Pacific bottlenose dolphin and the Indian Ocean humpback dolphin. Differences in the patterns of CT sound frequencies were observed at the study sites in the present study (Fig 6). A distinct peak at 119–122 kHz was recorded at all locations, corresponding closely to known *Tursiops sp*. and *Sousa sp*. broadband clicks. However, there was also a noticeable reduction in the prominence of this peak from Usine to the L’Oasis and Massoni sites. Conversely a small peak around 79–84 kHz recorded at Usine was dramatically increased at L’Oasis. Previous research indicates that *S*. *plumbea* make up a greater proportion of dolphin encounters near L’Oasis with *T*. *aduncus* occurring most commonly, in larger groups, near Usine [35]. Further, bycatch of *S*. *plumbea* has only been documented in the inshore bottom set gillnet fisheries near the L’Oasis site and to the north towards Massoni, whereas *T*. *aduncus* bycatch is almost exclusive to driftnet fisheries further offshore in the bay and towards the southern tip of Unguja Island [19,20]. In light of this and the lack of data regarding *T*. *aduncus* and *S*. *plumbea* broadband click vocalisations, changes in the pattern of CT sound frequencies suggest differences in vocal behaviour between the two species. Further investigation is required for comprehensive assessment of the specific echolocation behaviours of both MBCA species. This may enable future species separation and greatly improve the feasibility of PAM as a long-term monitoring solution in the area.

In a wider context this study adds to the body of work which highlight the need to understand the temporal variations in cetacean distribution and behaviour in order to create effective spatially selected conservation and management strategies. Static spatial strategies are likely insufficient or sub-optimal for organisms with expansive distribution and range of movement and management must be responsive to changes in distribution and habitat importance over time [63,64]. To date, only one such area shows evidence of increased odontocete survival [65]. Critical habitat protection approaches often fail to address shifts in habitat use throughout the diel cycle [66–70]. Time area closures or restrictions of human activities could help to maximise effectiveness of spatial measures. Populations in areas of high boat traffic and/or low enforcement capability would stand to benefit most. In the MBCA restricting net gear use and/or enforcing the use of bycatch mitigation technologies near Usine and L’Oasis during hours of darkness, when dolphin occurrence is highest, may be an effective way to reduce bycatch. The results from this study and other recent work [57] and anecdotal evidence suggest MBCA dolphin distribution shifts partially towards offshore reefs during daylight. Regulation of tourism and gillnet fishing in such areas, if identified, may need to be considered. Without baseline and historic data for distribution it is not possible to assess changes over inter-annual periods.

Critically, monitoring methodology must be consistent, high yield and cost effective and PAM techniques can provide this. Further development of reliable and, critically, time-efficient methods of species separation using acoustic data alone is therefore of upmost importance. Without this species specific patterns may be masked and the power of these monitoring programs would be compromised. The data and results from this study may serve as a starting point for monitoring dolphin occurrence in the MBCA. Continued and expanded monitoring of dolphin occurrence in the MBCA will be a vital component in future conservation efforts.

## Supporting Information

S1 Table CPOD.exe detection positive minute outputs for Massoni, L’Oasis and Usine. Detection Positive Minutes (DPM), Total Unfiltered Clicks (Nall) and Minutes On (MinOn) are displayed.
(XLSX)
